# The 3D Monolithically Integrated Hardware Based Neural System with Enhanced Memory Window of the Volatile and Non‐Volatile Devices

**DOI:** 10.1002/advs.202402667

**Published:** 2024-06-17

**Authors:** Yu‐Rim Jeon, Donguk Seo, Yoonmyung Lee, Deji Akinwande, Changhwan Choi

**Affiliations:** ^1^ Department of Electrical and Computer Engineering The University of Texas at Austin Austin Texas 78172 USA; ^2^ Department of Electrical and Computer Engineering Sungkyunkwan University Suwon 16419 South Korea; ^3^ Division of Materials Science and Engineering Hanyang University Seoul 04763 South Korea

**Keywords:** 3D neuromorphic system, CMOS integration, convolutional neural network, high k metal oxide, RRAM, synaptic device, transistor, wafer bonding

## Abstract

3D neuromorphic hardware system is first demonstrated in neuromorphic application as on‐chip level by integrating array devices with CMOS circuits after wafer bonding (WB) and interconnection process. The memory window of synaptic device is degraded after WB and 3 Dimesional (3D) integration due to process defects and thermal stress. To address this degradation, Ag diffusion in materials of Ta_2_O_5_ and HfO_2_ is studied in a volatile memristor, furthermore, the interconnection and gate metal Ru are investigated to reduce defective traps of gate interface in non‐volatile memory devices. As a result, a memory window is improved over 10^6^ in both types of devices. Improved and 3D integrated 12 × 14 array devices are identified in the synaptic characteristics according to the change of the synaptic weight from the interconnected Test Element Group (TEG) of the Complementary Metal Oxide Semiconductor (CMOS) circuits. The trained array devices present recognizable image of letters, achieving an accuracy rate of 92% when utilizing a convolutional neural network, comparing the normalized accuracy of 93% achieved by an ideal synapse device. This study proposes to modulate the memory windows up to 10^6^ in an integrated hardware‐based neural system, considering the possibility of device degradation in both volatile and non‐volatile memory devices demonstrated by the hardware neural system.

## Graphical abstract

Monolithically 3D integrated on‐chip neural system is demonstrated using CMOS circuits, considering the degradation of memory window after wafer bonding and interconnection processes. Ag diffusion in bi‐layer is studied in volatile memristors. Additionally, interconnection and gate metal are investigated to reduce defective traps in non‐volatile transistor. A memory window is improved over 10^6^ in both types of devices.

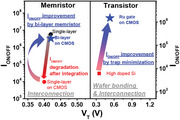

## 1 Introduction

Neuromorphic system has been attracting attention as an alternative computing system to the current von‐Neumann system thanks to low power consumption and capability to process non‐structured/structured data. A bottleneck generates between CPU and memory in von‐Neumann computing system when processing large amounts of data, however, neuromorphic system that is composed parallel structure like a biological neural network addresses input and output simultaneously. In addition, as the density‐down of the computing system faces a serious problem due to the limitations of scale‐down of semiconductor devices, the various approaches have been studied with materials, devices, neural network composition, and architecture to develop neuromorphic computing system. In order to mimic a neural network, various studies on artificial neural networks such as binarized neural networks,^[^ ^1^, ^2^ ^]^ Bayesian neural network,^[^ ^3^ ^]^ convolutional neural networks^[^ ^4^, ^5^, ^6^ ^]^ spiking neural networks.^[^ ^7^, ^8^, ^9^ ^]^ Moreover, demonstrating a hardware neuromorphic system goes beyond a single device and approaches it by connecting it to CMOS circuits in an array device. The array synaptic device was designed as transistor^[^ ^10^, ^11^, ^12^ ^]^ or memristor,^[^ ^13^, ^14^, ^15^, ^16^ ^]^ especially, Resistive Random Access Memory (RRAM) has been adopted to study low power neural network computation system for deep learning^[^ ^17^, ^18^, ^19^ ^]^ and neural networks^[^ ^20^, ^21^, ^22^, ^23^, ^24^, ^25^, ^26^ ^]^ thanks to its simple structure with metal‐insulator‐metal, small device dimensions, and CMOS compatibility for on‐chip systems. Therefore, by utilizing the strengths of RRAM, the on‐chip system was demonstrated for a short‐term memory system,^[^ ^27^ ^]^ compute‐in‐memory^[^ ^28^ ^]^ or vertically integrated photo‐detecting system.^[^ ^29^ ^]^ Moreover, the RRAM array was vertically deposited and interconnected on the back‐end‐of‐line (BEOL) of a CMOS wafer in previous studies.^[^ ^30^, ^31^, ^32^ ^]^ The thin‐film‐transistor (TFT) device was also studied to integration on BEOL.^[^ ^33^, ^34^, ^35^ ^]^ This vertical integration or packaging method is considered necessary not only for neuromorphic systems but also for typical computing systems. The human brain efficiently processes and transmits complex data using a neural network comprising ≈10^11^ neurons and 10^15^ synapses, characterized by intricate connections, high integration, and the parallel processing of vast amounts of information with minimal power consumption. To address the growing complexity of big data, the study of vertically integrated neuromorphic systems is required. A longer signal delivery distance in the 2D methodology is inevitable due to routing density increasing according to the number of connections, resulting in an increase in die area and power consumption.^[^ ^36^ ^]^ However, previous studies had a fragmentary limitation that only focused on demonstrating a vertical neuromorphic system that can be applied to RRAM devices^[^ ^36^ ^]^ or focused on the model verification of an interconnection with the neural circuit.^[^ ^37^ ^]^ Another previous paper developed a synaptic transistor with III−V channel materials that could be integrated in 3D using wafer bonding, but it was not possible to expand it to the system.^[^ ^38^ ^]^ Therefore, for demonstrating 3D hardware neuromorphic system, overall studying system design, device fabrication and integration, and verifying electrical property are demanded.

In this study, for the first time, a 3D hardware on‐chip neuromorphic system was successfully demonstrated and realized by interconnecting neural CMOS circuits and synapse array devices with volatile and non‐volatile memory devices after WB, while improving synapse memory window over 10^6^. The vertically connected neuromorphic system, suitable for the current Si‐technology, consisted of a 12 × 14 synapse array RRAM that can be fabricated over a large area. The devices emulating synaptic plasticity follow synaptic weights via designed TEGs with pre‐synaptic CMOS circuits that generated spikes and selectively transmitted the spikes. The non‐volatile array devices of TFT structure were integrated with a fab‐outed CMOS wafer after WB and transfer. This 3D neuromorphic system well emulated the synaptic plasticity following the CMOS spikes and the accuracy of a pattern recognition was 92% accurate for a given image using a CNN. The demonstrated system was confirmed that could be vertically applicable any type of array synaptic devices and could be implemented as on chip state for verifying the electrical synaptic properties.

## 2 Results and Discussion

### 2.1 Design of Volatile Memristor for Integrated Neural System

**Figure 1:**
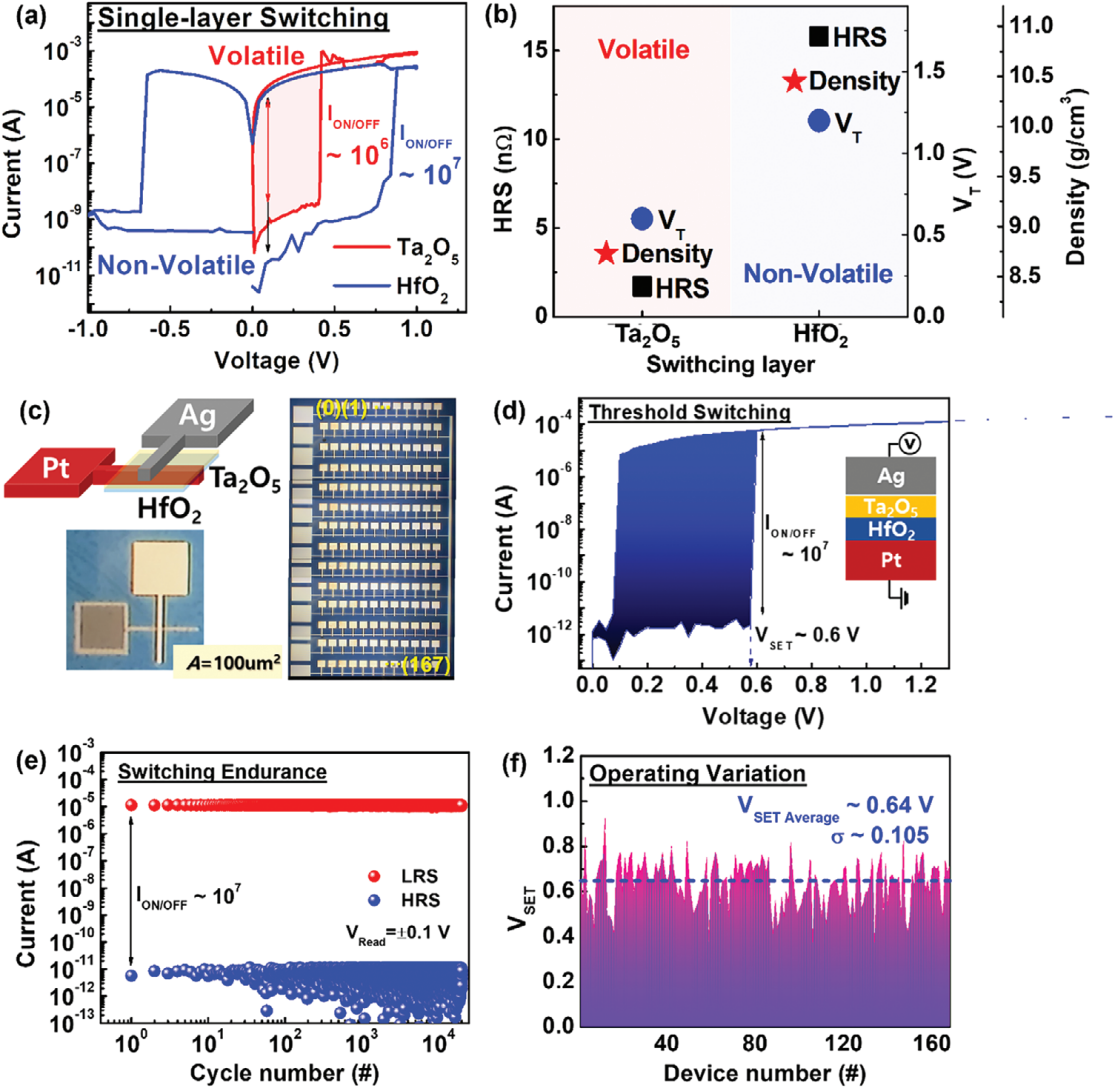
Schematic and characteristics with RRAM devices on Si substrate. a) Single‐layer devices of Ta_2_O_5_ and HfO_2_ exhibit both volatile and non‐volatile switching. b) Comparison of HRS, V_T_, and film density with Ta_2_O_5_ and HfO_2_ film, respectively. c) Structure of the RRAM device consists of a 100 µm^2^ array with an Ag/Ta_2_O_5_/HfO_2_/Pt layer sequence and an optical microscope image of the 168 arrays. d) The I‐V curve and e) pulse endurance of RRAM. f) Reliability characteristics of the 168 arrays with Ag/Ta_2_O_5_/HfO_2_/Pt corresponding V_SET_ variation.

For demonstrate the volatile neural system, Ta_2_O_5_, and HfO_2_ high‐k metal oxide materials were studied by identifying property as shown in **Figure** **1**a,b. The Ta_2_O_5_ device exhibited volatile memory characteristics with an I_on/off_ ratio of 10^5^, while the HfO_2_ device demonstrated non‐volatile characteristics, with the HfO_2_ film showing a relatively high film density and high resistance state (HRS) of ≈15.8 nΩ and 10.45 g cm^−3^, respectively, compared to the Ta_2_O_5_ film. Ag‐based volatile memristor operates as following Ag atom migration in the intermediate layer, which could be designed the characteristics of memory window, its volatile and non‐volatile. Ta_2_O_5_ film could enhance the Ag metal diffusion mobility following oxygen vacancy due to Schottky defect compared to HfO_2_ film. To enhance device endurance and increase the I_on/off_ ratio, a bilayer device with Ta_2_O_5_ and HfO_2_ was designed and fabricated in a 12 × 14 array on a Si substrate, covering a total area of 100 µm^2^ and denoted by device numbers ranging from 0 to 167 in Figure 1c. As shown in Figure 1d, the designed bilayer device showed volatile property with a low operating voltage of 0.6 V and I_on/off_ ratio of ≈10^7^. Considering the potential degradation of memory characteristics during the integration process, the design of the short‐term memory synapse device aimed to maintain a memory window of 10^7^ or more even during repeated on/off cycles, which was an improvement compared to a memory window of 10^6^ in single Ta_2_O_5_ devices.

When the structure was designed as bilayer and applied with the bias, oxygen vacancies in an interface increased resulting Ag diffusion, which made the memristor volatile property. This volatile SET behavior was significantly influenced by the filament size and the metal ion surface energy within switching medium and the filament breakage was expected when filament width was below a certain threshold width. Small Ag ion particles with high curvature tend to dissolve for a reduction of the effective surface energy since nucleation is thermodynamically favorable for the smallest surface energy.^[^ ^39^, ^40^ ^]^ Therefore, a conductive filament became irretentiveness due to the diffusion of Ag atoms. When a voltage above the threshold was applied, the Ag atoms diffused in the insulator layer and the conductive filament formed, which was low resistance state (LRS). However, when the voltage decreased below the threshold, the conductive filament could not be maintained and Ag atom was diffused following the diffusion equation due to the surface energy of the Ag filament, which became a high resistance state (HRS). The device reliability was investigated as in Figure 1e,f, which appeared the endurance of switching cycle above the 10^4^ with maintaining the I_on/off_ ratio ≈10^7^. For 168 devices, the SET voltage variation device‐to‐device was measured and was obtained ≈0.105 and the average V_SET_ was ≈0.64 V.

### 2.2 Verifying Volatile Memristor Interconnected with CMOS Circuits

**Figure 2:**
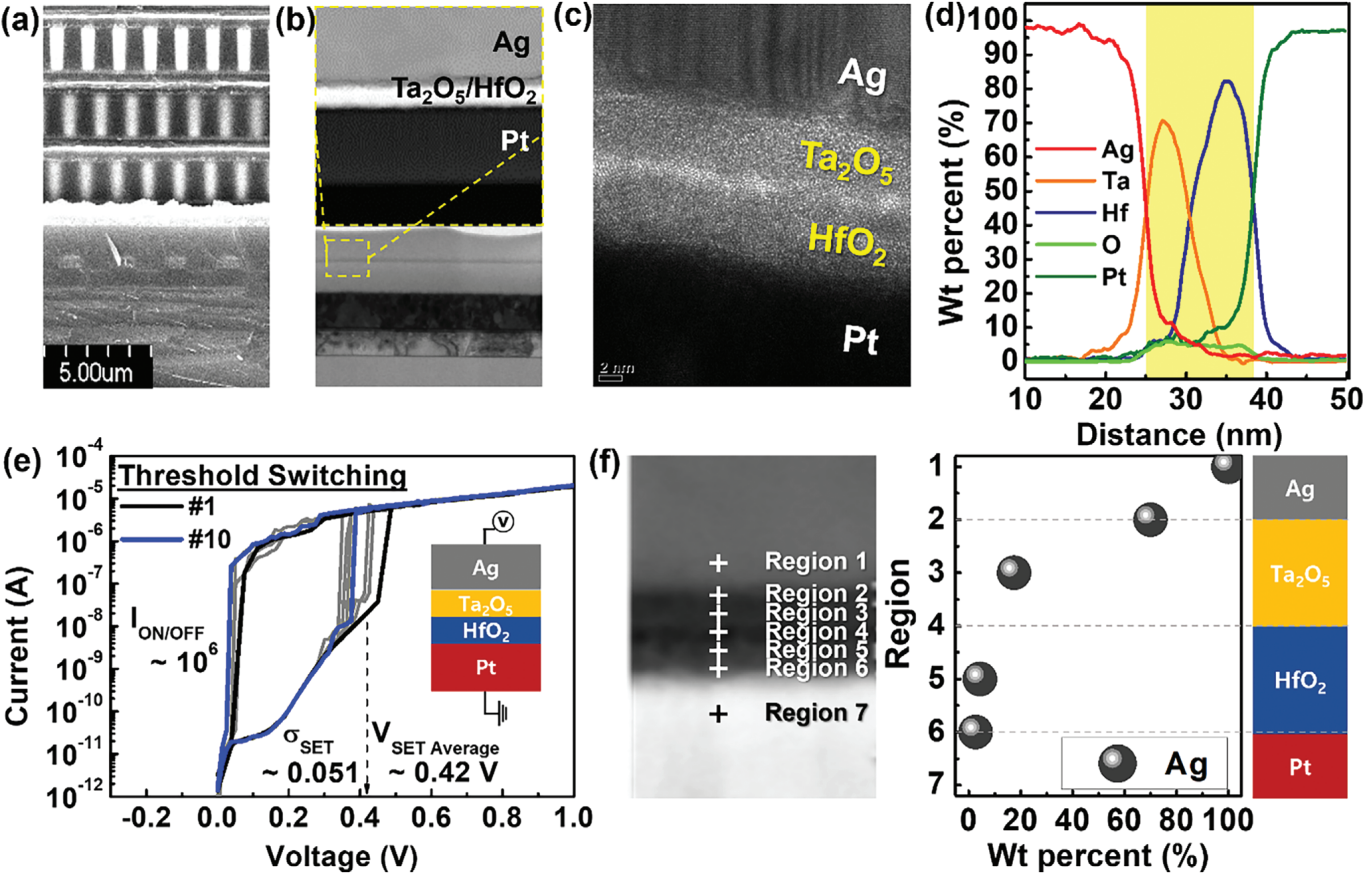
Cross‐sectional images, electrical and chemical property of volatile array devices interconnected with CMOS circuits. a) The cross‐section view of CMOS wafer and b) high magnification images of RRAM on bottom of CMOS wafer. c) STEM image with structure Ag/Ta_2_O_5_/HfO_2_/Pt and d) EDS mapping of Ag, Hf, O, and Pt elements. e) The electrical characteristics of the volatile device incorporated CMOS. f) The point EDS analysis along the cross‐sectional layers from region 1 to region 7 after applying V_SET_ to the device.

**Figure 3:**
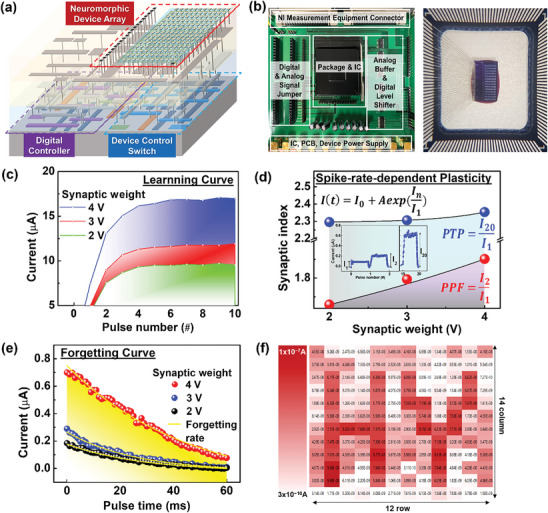
Synaptic plasticity of the neuromorphic system with the volatile array device interconnected to CMOS circuits. a) A schematic image of neuromorphic system interconnected CMOS TEG. b) PCB board and packaged array devices for on‐chip level evaluation. c) A learning curve according to the synaptic weight 2–4 V and 10 ms duration. d) Short‐term memory characteristics of PPF and PTP. The fraction values of I_1_ and I_2_ or I_1_ and I_20_ were calculated from learning curve in (a), which represented EPSC level after excitatory pre‐synaptic spikes. e) A forgetting curve according to the synaptic weight 2–4 V. f) The training image of “H” and “Y” characters on 12 × 14 array device. The current level was measured after applying ten training pulses at 3 or 4 V with a pulse duration of 0.5 ms.

The validated short‐term synapse devices were interconnected using the designed photolithography mask in back‐end to CMOS circuits as **Figure** **2**a,b, which included a pre‐synapse designed to generate and selectively transmit pulses to the synapse array. STEM (scanning transmission electron microscopy) image and cross‐sectional EDS (energy‐dispersive X‐ray spectroscopy) analysis were used to verify the volatile memory devices deposited on the CMOS circuits, as shown in **Figure** **3**c,d. The STEM and EDS analysis revealed the elemental composition of the Metal‐Insulator‐Metal (MIM) structure, which consisted of Ag, Ta, Hf, O, and Pt elements.

The I‐V characteristics of 12 × 14 array devices on the CMOS show V_SET Average_ of ≈0.42 V, I_on/off_ ratio of ≈10^6^ and a stable volatile switching during the ten repeated cycles as shown in Figure 2e. Compared with the devices on a silicon substrate, the devices integrated with the COMS circuit showed a degraded memory window from 10^7^ to 10^6^ of I_on/off_ ratio. For demonstrating 3D neuromorphic system with volatile synapse device, it is important considering degradation of the memory. Therefore, the volatile synapse device was designed to possess a substantial memory window of 10^7^ alongside its volatile characteristics, and it consistently displayed stable switching characteristics with a 10^6^ of I_on/off_ ratio during repeated 10 cycles. The variation of an average of SET voltage was ≈0.051.

To confirm the Ag diffusion mechanism in the bilayer, point EDS analysis was performed along the cross‐sectional layers after applying V_SET_ to the device. Figure 2f shows seven distinct points within the region and presents the Ag weight percentage in each area. The results demonstrated that the Ag concentration was 100% in the Ag layer of region 1 and gradually decreased within the Ta_2_O_5_ and HfO_2_ layers. It is worth noting that at the interface of Ta_2_O_5_ and HfO_2_, the Ag concentration was 0% due to the controlling effect of the Ta_2_O_5_ layer, which enhanced Ag diffusion, thereby stabilizing the volatile switching properties. By controlling the Ag diffusion mechanism, the array devices can exhibit various synaptic behaviors, which are essential for mimicking the biological synaptic plasticity in neuromorphic systems.

### 2.3 Synaptic Plasticity of Volatile Memristor‐Based Neuromorphic System

The incorporated RRAM array with CMOS circuits was packaged and attached to PCB for evaluating the synaptic properties of the on‐chip state neuromorphic system that controlled the generating pulses and connected them to specific devices in the array as shown in Figure 3a,b. Using the neuromorphic system, we obtained synaptic behavior according to the Hebbian learning rule that the post‐synaptic current level also increases when the strength of the synaptic weight increases. As the synaptic weight increased from 2 to 4 V, the gradient of curve fitting, which represented the learning rate, also increased from 9.5 to 17 µA/# in Figure 3c, similar to a biological synapse in the human brain. In learning and memory processes of the brain, SRDP is a type of synaptic plasticity that is dependent on the rate of action potentials or spikes that occur in a pre‐synaptic neuron, which refers to the phenomenon where the strength of a synapse is increased when the pre‐synaptic neuron fires at high rates, and decreased when it fires at low rates. The neuromorphic system was sufficient to demonstrate the memorization ability of a biological brain system, with an array device integrated into CMOS circuits that acted as a pre‐synapse.

Based on the learning characteristics, the PPF and PTP were calculated by fraction value of excitatory post‐synaptic current (EPSC) level in I_1_ and I_2_ or I_1_ and I_20_ of the learning curve as shown in Figure 3d. The PPF and PTP properties were a form of short‐term memory characteristics, which draw exponential function as synaptic weight increase.

(1) $$I\left(t\right)=I_{0}+Aexp\left(\frac{I_{n}}{I_{1}}\right)$$

where I(t) was the current function at the current I_1_, and I_n_. The A and I_0_ were the constant values. The synaptic index growth rate increased as the synaptic weight from 2 to 4 V.

After strong stimulation, the synaptic current levels were read by the applying read voltage in Figure 3e, which was forgetting curve following Equation (3).

(2) $$I^{\prime }\left(t\right)={I^{\prime }}_{0}+A^{\prime }exp\left(-\frac{t}{\tau }\right)$$

where I’(t) and I’_0_ were the current relaxation function at time t, A’ was the constant value, and τ was the relaxation time. The decreasing rates of the exponential formula were ≈8.9 × 10^−7^, 2.9 × 10^−7^, and 2.0 × 10^−7^, respectively. However, for a stronger stimulus from 2 to 4 V, it took longer to return to the initial state. The filled areas of Figure 3c,e were the amount of change in the synapse level, and the greater the synaptic weight applied, the greater the change in the synapse level.

Finally, the synapse 12 × 14 array was trained with strong 4 V learning weights for the words “**H**” and “**Y**”, while the rest of the devices were trained with 3 V synaptic weights. After applying 10 pulses and waiting for a 0.5 ms relaxation time, a current level was measured at a read voltage, and the level showed a difference according to the trained synaptic weights in a mapping image of Figure 3f. Except for some elements, it was confirmed that the letters “**H**” and “**Y**” were clearly learned. These results showed that the integrated volatile neural system with CMOS circuits demonstrates a biological neural system.

### 2.4 3D Integrated Neural System with Non‐Volatile Devices after Wafer Bonding Process

**Figure 4:**
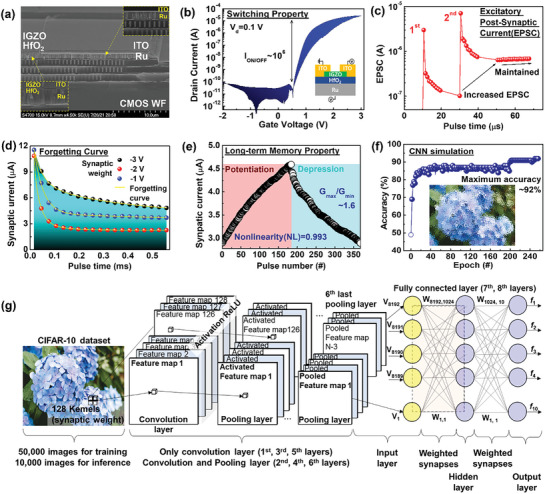
The incorporated non‐volatile array device with CMOS circuits. a) A cross‐sectional image of inter‐connected TFT with bottom of CMOS after wafer bonding and transfer. b) I‐V switching properties of an inserted device structure. c) EPSC change of a synaptic weight 3 V and 20 µs. d) Forgetting curve according to the synaptic weight from −1 to −3 V and 10 µs duration. e) Potentiation and depression synaptic properties. f) Accuracy of the neuromorphic system with the non‐volatile synapse devices according to epoch with CNN simulation. g) Schematic illustration of the CNN architecture. CNN simulation with an integrated neural system using non‐volatile synapse devices.

A non‐volatile synapse memory was designed as an array with a back gate as shown in Figure S1 (Supporting Information) using Ru for the interconnection metal. Compared to a transistor with a highly doped Si gate, the transistor exhibited improvements in the I_on/off_ ratio, which is associated with the memory window. The memory window and I_on/off_ ratio can be impacted by interface between gate metal and HfO_2_ insulator and may experience degradation. Due to gate patterning and Ru metal gate electrode, the I_on/off_ ratio increased from 10^4^ to 10^6^. After pre‐treating the bonding surface with N_2_ and O_2_ plasma, the direct WB process was carried out using an 8‐inch Silicon‐On‐Insulator (SOI) wafer and a CMOS wafer. The Si layer of SOI was transferred to a surface of CMOS wafer and Ru interconnection and back gate were filled and deposited for demonstrating synapse transistor as shown in **Figure** **4**a.

The memory window was attributed to charge carrier trapping and de‐trapping near the channel, and the threshold voltage (V_th_) was shifted due to the influence of trapped and de‐trapped charges.^[^ ^41^, ^42^, ^43^, ^44^, ^45^ ^]^ A on current increased as the increased V_d_ from 0.1 to 0.8 V during the applied gate bias sweep as shown in Figure S1 (Supporting Information). The on‐chip non‐volatile neural system using the transistor array device was investigated by applying the pulses generated and controlled in the CMOS circuits, pre‐synapse to the integrated devices.

To ensure systemic verification with a 3D integration, the wafer bonding and transfer process were studied and applied to the neuromorphic system. To prevent damage of CMOS circuits, the wafer bonding process was carried out in low temperature 400 °C. After pre‐treating the bonding surface with N_2_ and O_2_ plasma for conversion to hydrophilic surface,^[^ ^46^, ^47^ ^]^ the direct WB process was carried out using an 8‐inch SOI wafer and a CMOS wafer. The Si layer of the SOI was transferred onto the surface of the CMOS wafer through substrate etching, and Ru was deposited as the interconnector and back gate electrode to demonstrate the synapse transistor as shown in Figure 4a.

As the device scale was decreased, Ru was found to have high electrical conductivity ≈1.4 × 10^7^ S m^−1^, making it a suitable material for interconnection purposes.^[^ ^48^, ^49^ ^]^ The use of low‐resistance interconnects is important for reducing power consumption and ensuring efficient signal propagation within the neuromorphic system. By utilizing Ru as an interconnection material, the overall power consumption of the system can be minimized, while still maintaining high performance and reliability. The vertically integrated transistor exhibited an I_on/off_ ratio of 10^6^, which was similar to the pre‐integration value in Figure S1 (Supporting Information) and Figure 4b. Due to the modulation of the gate metal with Ru, the transistor was able to achieve a non‐volatile memory window of 10^6^ after WB and CMOS integration. The on‐chip non‐volatile neural system using the transistor array device was investigated by applying the pulses generated and controlled in the CMOS circuits, pre‐synapse to the integrated devices. The post‐synaptic current level increased from an initial 3 to 7.1 µA and persisted after two pairs of synaptic weights (3 V/20 µs) were transmitted from the pre‐synapse CMOS circuits in Figure 4c. In brain science, an excitatory postsynaptic potential (EPSP) makes the post‐synaptic neuron more likely to fire an action potential. This is caused by an increase in the flow of positively charged ions, resulting in an EPSC.^[^ ^50^ ^]^ It was confirmed that the synaptic neurons following the brain science were sufficiently imitated by utilizing the neuromorphic system at on chip state. After strong stimulation from −1 to −3 V was applied to the synapse device, the synaptic current level was measured, and it appeared that the current level decreased, showing forgetting properties, as shown in Figure 4d. The rates of decrease calculated by the exponential formula were ≈6.52 × 10^−6^, 1.1 × 10^−5^, and 1.55 × 10^−5^, respectively, and it was observed that the rate of forgetting decreased when strong stimulation from −1 to −3 V was applied. In the psychology of learning, Atkinson and Shiffrin studied the multistore model of human memory.^[^ ^51^, ^52^ ^]^ Memorization involves a 3‐step process: sensory memory, which stores information for a very short time, short‐term memory, which stores information temporarily, and long‐term memory, which stores information permanently. New information can transition into long‐term memory through the process of rehearsal, depending on the frequency and strength of the stimulus. Similar to human learning, synaptic transistor devices exhibited learning properties when the stimulus strength from −1 to −3 V was stronger in the pre‐synaptic CMOS circuits. The demonstrated transistor neuromorphic array device had been reliably and continuously trained using the neuromorphic system, which is shown in Figure 4e for linear potentiation and depression. The maximum rate of synaptic conductance G_max_/G_min_ was ≈1.6 and the nonlinearity (NL) was defined following.^[^ ^53^ ^]^

(3) $$NL=\frac{max\left|G_{p}\left(n\right)-G_{d}\left(n\right)\right|}{G_{p}\left(max\right)-G_{p}\left(min\right)}for\;n=1∼183$$

where G_p_(n) and G_d_(n) are the conductance values after the n^th^ pulse of potentiation and depression. The calculated NL value was ≈0.993, which is close to 1, showing a high linearity value. The obtained potentiation and depression properties were simulated using the CIFAR‐10 dataset with training data of 50 000 images and inference data of 10 000 images, as shown in Figure 4f,g. The neural network with ReLU activation was composed of the 6th convolutional layer, the 3rd pooling layer, and the 2nd fully connected layer. The accuracy rapidly increased over the 85% in the initial step of epoch as shown in Figure 4f. Finally, the integrated neuromorphic system achieved a high accuracy of ≈92%. This accuracy rate can be compared to the normalized accuracy of 93% achieved by an ideal synapse device with a nonlinearity 1. It has been confirmed that the proposed system can emulate a long‐term neural system. Moreover, the proposed neuromorphic system has been confirmed to be capable of integrating and utilizing all types of neuromorphic devices while maintaining stable electrical properties and a standardized framework for the 3D integrated neural system was provided for the implementation of the hardware neural system and its subsequent on‐chip level evaluation.

## 3 Conclusion

**Figure 5:**
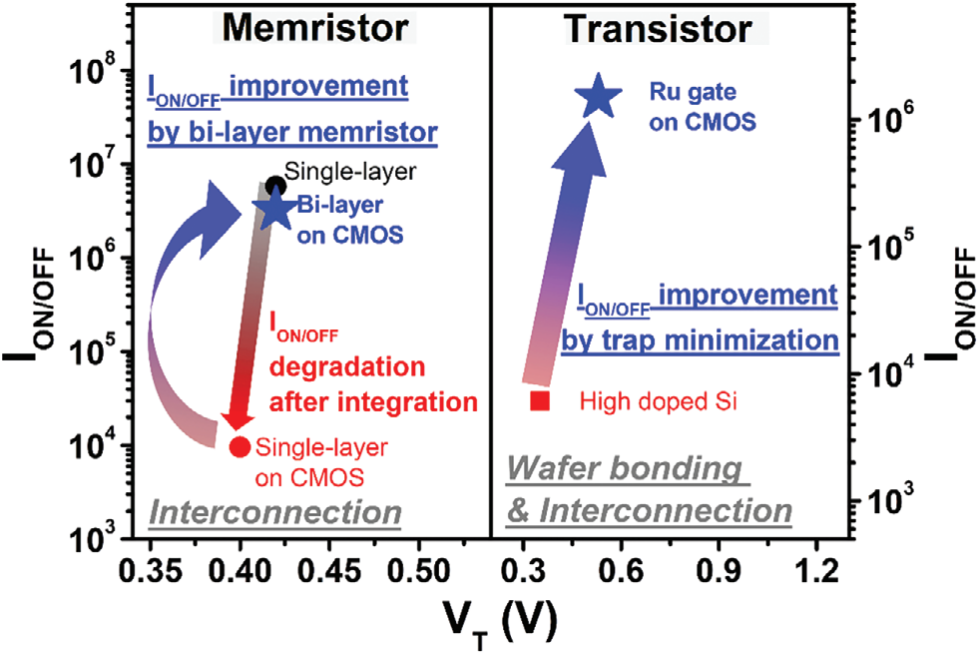
Monolithically 3D integrated on‐chip neural system for hardware‐based platform. Experimentally identified process window of memory device property for integrated neuromorphic system.

A monolithically 3D integrated on‐chip neural system was demonstrated using CMOS circuits, considering the degradation of synaptic memory window after wafer bonding and interconnection processes. To improve the device memory properties, the memristor array was studied and designed as a volatile synapse with a Ta_2_O_5_ and HfO_2_ bi‐layer, while the transistor array incorporated a Ru gate after the wafer bonding and interconnection filling process. As a result, the integrated volatile memristor array exhibited the memory window over 10^6^, durability reliability over 10^4^, and 0.088 values of σ in the device‐to‐device operation change. Additionally, the non‐volatile transistor array demonstrated a 10^6^ of I_on/off_ ratio. The designed CMOS circuits acted as a pre‐synapse, stimulating the integrated synaptic memory array. It was trained in an on‐chip state to exhibit short‐term memory properties using volatile devices and long‐term memory properties using non‐volatile devices. In particular, obtaining recognizable H and Y image and a 92% accuracy of the Convolution Neural Network (CNN) simulation, which has been verified to reliably mimic the biological neural properties. The constructed neuromorphic system was verified to be capable of reliably modulating biological neuromorphic properties using all types of devices. The demonstrated neuromorphic system was verified to be reliable in the 3D vertically stacking process, which is important for the scale‐down semiconductor industry and achieving an on‐chip state. As a results, a standardized framework for the 3D integrated neural system is shown in **Figure** **5** for the implementation of the hardware neural system and its subsequent on‐chip level evaluation. When the developed neuromorphic system was compared with the published results, it was observed that the on‐chip level CMOS integrated neuromorphic system, applicable to any type of neuromorphic device, was demonstrated with both volatile and non‐volatile synaptic arrays, as shown in **Table** **1**. This hardware neuromorphic system has the potential to be applied in various semiconductor industries for artificial intelligence computing.

**Table 1:** Comparison of hardware based neuromorphic system. Note that the on‐chip level 3D integrated neuromorphic system is only this work.

	Ref. [10]	Ref. [11]	Ref. [12]	Ref. [13]	This work
On‐chip evaluation	X	O	X	O	O
3D integration (with WB)	X	X	X	X	O
Integrated synapse device	RRAM	RRAM	RRAM	RRAM	RRAM Transistor
Memory window (I_ON/OFF_)	≈10^5^	≈10^3^	≈10	≈10^3^	RRAM ≈10^6^ Transistor ≈10^6^
Simulation accuracy	SNN 86%	Edge NN 99%	DNN 98%	MNIST 89%	CNN 92%
This work	✓ The first time 3D integrated hardware neural system with both volatile and non‐volatile synapse array.				

## 4 Experimental Section

### Neuromorphic System and CMOS Circuits

The developed neuromorphic system was focused on the demonstrating a biological neural system, which spikes over the threshold transmit to end of the neuron and stimulate a synapse that could stimulate another neuron. Synapses that are frequently stimulated are strengthened, or synapses that are less stimulated are not well connected, which is synaptic plasticity that is the mechanism of memorization. The aim of the system is to emulate this behavior of biological neural system in order to develop more efficient and adaptable hardware computing systems. A hardware neuromorphic system was composed of CMOS circuits generating the spike and synapse array device interconnected with CMOS as shown in Figure S2a (Supporting Information). The mask layouts were designed for CMOS circuits and array device and were appeared in a dotted highlight of Figure S2a (Supporting Information). CMOS circuits were designed as the TEG with digital controller and control switch, which generated and derived the controllable pulses to automatically selected neuromorphic devices in Figure S2b (Supporting Information). The digital controller was responsible for pulse tuning by controlling the pulse amplitude, pulse width, pulse frequency, and pulse number. The generated positive or negative pulses were delivered to a control switch and applied to a device that was opened by a control switch. This enables the TEG to activate specific neuromorphic devices with precise timing and control, allowing for the emulation of biological neural networks. The use of digital control also ensures that the circuit can be easily programmed and modified to achieve specific network behaviors. TEG was implemented using CMOS devices based on 180 nm technology. After the CMOS wafer designed TEG was fab‐outed, the synaptic array devices were demonstrated with two and three terminal structures were integrated onto the CMOS wafer. This was achieved using via etching and filling with Ru. To demonstrate a low‐power neuromorphic system, Ru was used as an interconnection metal for the synaptic array devices integrated onto the CMOS wafer. In particular, the synapse array was fabricated and interconnected on a SiO_2_/Si layer that was transferred layer from a SOI wafer using the wafer bonding process.

### Synapse Devices Fabrication

As synaptic device, the array devices were formed with RRAM and TFT structures on the CMOS wafer. RRAM structure with an Ag/HfO_2_/Pt array was fabricated with a size of 12 × 14 by using the DC sputter, Atomic Layer Deposition (ALD), and thermal evaporator, respectively. A thin film deposition technique, ALD that could control the deposition thickness by atomic scale^[^ ^54^ ^]^ was used because the inter‐layer HfO_2_ of RRAM was crucial for the device characteristics that mimicked synaptic properties. The three terminal structured TFT with Indium‐Gallium‐Zinc Oxide (IGZO) channel was deposited on the high‐k/metal gate stack of the HfO_2_/Ru. The electrode for source and drain (S/D) was an ITO metal deposited by DC sputtering. As shown in Figure S3 (Supporting Information), the fabricated synapse array devices on the CMOS wafer were diced with a size of ≈1.8 mm × 1.8 mm and packaged with PPGA. Finally, a packaged chip containing the integrated synapse devices was attached to a PCB board for synaptic characteristic evaluation, as shown in Figure S3 (Supporting Information). The yellow highlight box in Figure S3b,c (Supporting Information) represents a high magnification of the package and IC, which showed the die‐to‐die wire bonded sample.

### Electrical Measurement Scheme

The measurement setup of the proposed TEG was consisted of a power supply (2231A‐30‐3), PCB board, and NI equipment (NI‐6289). The power supply supplied power to the PCB board and inside the IC chip. The PCB board was designed to consider integrated circuit (IC), measurement equipment, stable power supply, and power delivery network (PDN) to reduce power supply noise. The PDN was interconnected with the power supply path from the voltage regulator modules to the circuit of the IC, including all capacitors related to the power and ground planes of the layer, cables, and parasitic components. In addition, NI 6289 created a digital input vector with the Labview program. It had established a test environment in which digital input vectors control the pulse tuning controller of TEG to automatically control pulse amplitude, pulse width, pulse frequency, pulse number, and device array switch.

## Conflict of Interest

The authors declare no conflict of interest.

## Supporting information

Supporting Information

## Data Availability

The data that support the findings of this study are openly available in Jeon at https://doi.org/10.1021/acsami.3c13159, reference number 54.
