# Supplementary material for: The 3D Monolithically Integrated Hardware Based Neural System with Enhanced Memory Window of the Volatile and Non‐Volatile Devices
Source: Adv Sci (Weinh). 2024 Jun 17;11(31):2402667. doi: 10.1002/advs.202402667 (PMC11336934; doi:10.1002/advs.202402667)
Supplement: Supplementary file 1 — Supporting Information [file ADVS-11-2402667-s001.docx]

Supporting Information

The 3D Monolithically Integrated Hardware Based Neural System with Enhanced Memory Window of the Volatile and Non-volatile Devices

Yu-Rim Jeon^1^, Donguk Seo^2^, Yoonmyung Lee^2^, Deji Akinwande^1^* and Changhwan Choi^3^*

Dr. Y.-R. Jeon, Prof. D. Akinwande

Microelectronics Research Center, The University of Texas at Austin, Austin, Texas 78758, United States

D. Seo, Prof. Y. Lee

Department of Electrical and Computer Engineering, Sungkyunkwan University, Suwon 16419, Korea

Prof. C. Choi

Division of Materials Science and Engineering, Hanyang University, Seoul 04763, Korea

E-mail: [deji@ece.utexas.edu](mailto:deji@ece.utexas.edu), [cchoi@hanyang.ac.kr](mailto:cchoi@hanyang.ac.kr)


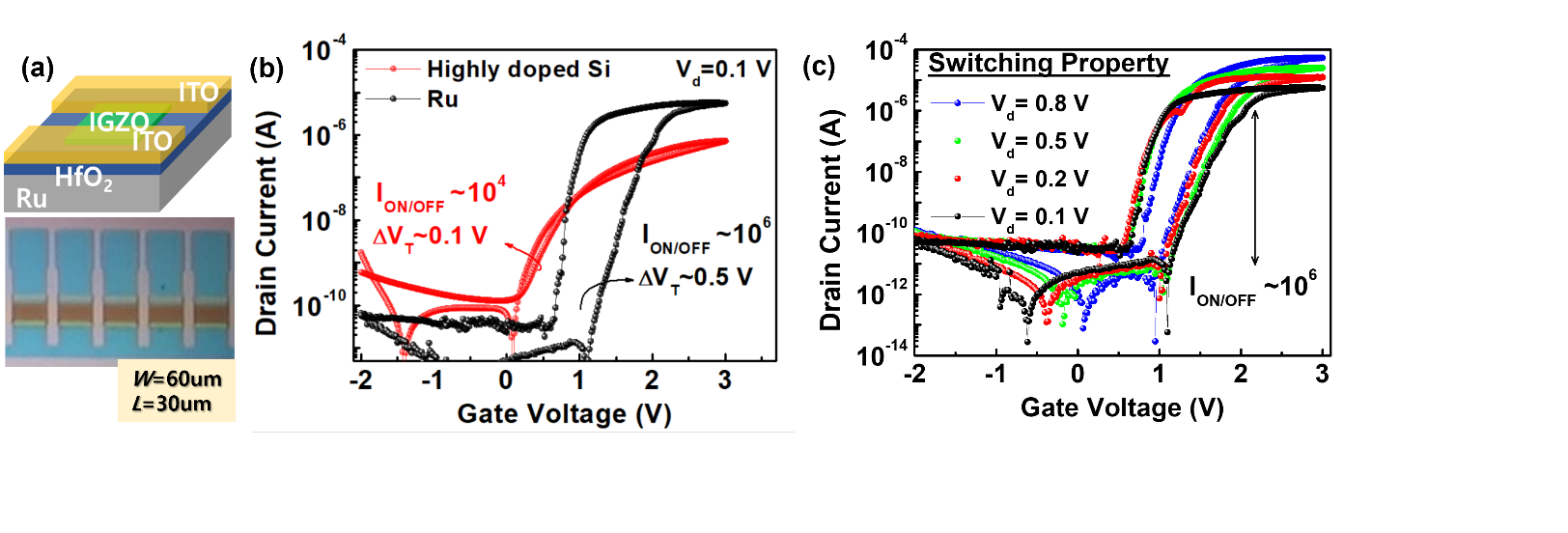


**Figure S1.** Schematic and characteristics with non-volatile synapse transistor array devices. (a) Schematic and optical images of ITO/IGZO/HfO_2_/Ru synaptic array devices. (b) The I-V characteristics of device switching with highly doped Si and Ru gate. Note that the I_ON/OFF_ ratio of the Ru gate improved by over 10^2^ compared to that of the poly Si gate. (c) The I-V characteristics of non-volatile device switching exhibit variations depending on the drain voltage, V_d_.


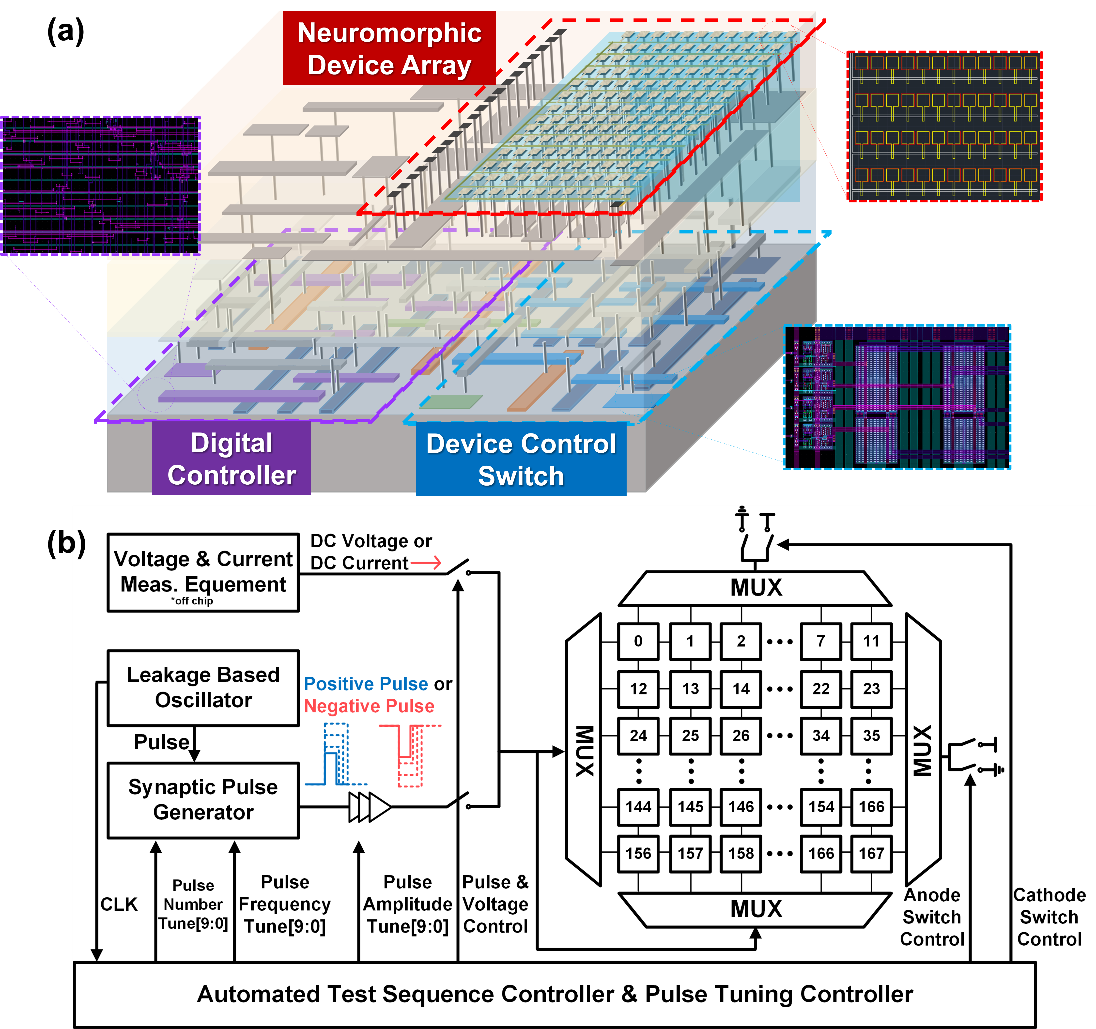


**Figure S2.** Vertical neuromorphic system integrated with CMOS circuitry. (a) Schematic and mask design diagrams of the neuromorphic system integrated in vertical direction. (b) The TEG design diagram of designed digital controller, pulse generator and control switch.


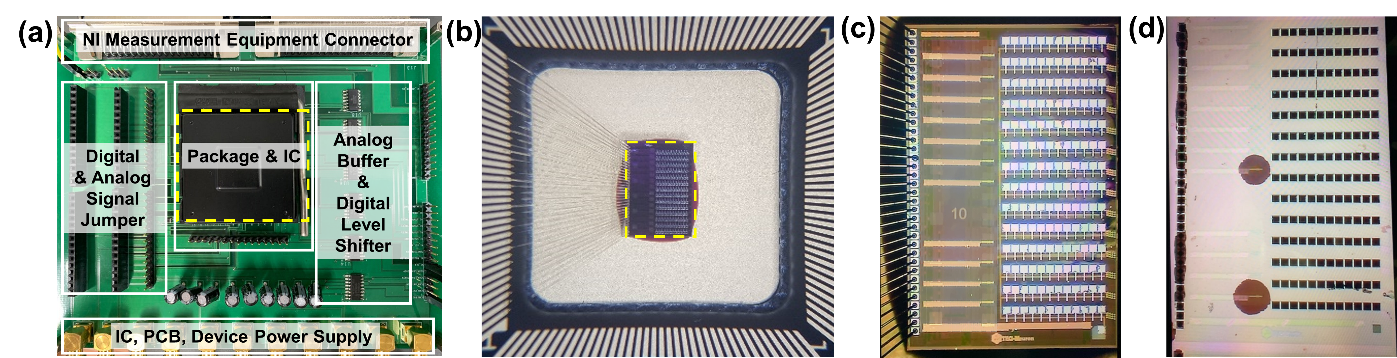


**Figure S3.** Measurement PCB design with packaged 3D integrated devices array and micrograph. The yellow highlight images are (a) The PCB board consisted of signal jumper, shifter, power supply, NI connector, and package & IC. (b) The high magnification image of the package & IC. The sample was diced and wired. (c) The high magnification of packaged sample. (d) Optical microscope image of the 3D integration system with non-volatile synapse array devices and CMOS circuits after wafer bonding process.
